# Impact of antibiotic pretreatment on cultures in children with osteomyelitis and septic arthritis: a retrospective review

**DOI:** 10.1186/s12887-021-02806-w

**Published:** 2021-08-13

**Authors:** Amanda Lansell, Yasasvi Vasili, Parminder S. Suchdev, Janet Figueroa, Anjali Kirpalani

**Affiliations:** 1 Children’s Healthcare of Atlanta, Atlanta, Georgia; 2 Emory University Department of Pediatrics, Atlanta, Georgia; 3 Rainbow Babies and Children’s Hospital, Cleveland, OH USA; 4 Present Address: Department of Pediatrics, Rainbow Babies and Children’s Hospital, 11100 Euclid Avenue, Cleveland, OH 44106 USA

**Keywords:** Child, Hospitalized, Arthritis, Infectious, Osteomyelitis, Anti-bacterial agents, Retrospective studies

## Abstract

**Background:**

In the management of pediatric osteomyelitis or septic arthritis, delay in treatment may affect outcome, while receipt of antibiotics prior to culture may affect culture results. We aimed to determine if pathogen identification decreased in cultures that were pretreated with antibiotics.

**Methods:**

We conducted a retrospective cohort study of 584 hospitalized children between 30 days and 18 years of age admitted to two tertiary children’s hospitals. Logistic regression assessed the effect of antibiotic duration on blood, bone, joint aspirate, and “other” culture positivity.

**Results:**

Overall, 42% of blood cultures, 70% of bone cultures, 39% of joint cultures, and 70% of “other” cultures were positive. Compared with children who did not receive antibiotics prior to culture, there were no significant differences in odds of a positive culture in children whose cultures were pretreated with antibiotics for any of the culture types [OR (95% CI) 0.90 (0.56–1.44) for blood cultures, 0.77 (0.25–2.34) for bone cultures, 0.71 (0.39–1.28) for joint cultures, 1.18 (0.58–2.41) “for other” cultures; all *p* > 0.05]. Furthermore, the duration (hours) of antibiotics in the pretreated cultures was also not a significant predictor of culture positivity (OR ranged from 0.99–1.00 for all cultures, *p* > 0.05).

**Conclusions:**

Culture positivity was not associated with antibiotic pretreatment in any of the samples, even for longer duration of antibiotics prior to culture, though the small sample size of subgroups is an important limitation. In pediatric patients hospitalized with osteomyelitis and/or septic arthritis, early initiation of antibiotics may not affect culture positivity.

**Supplementary Information:**

The online version contains supplementary material available at 10.1186/s12887-021-02806-w.

## Background

Osteomyelitis and septic arthritis are two diagnoses commonly encountered in pediatric hospital medicine with an incidence of 1 in 5000-10,000 [1–3] and 1 in 2700-100,000 [4], respectively. These diagnoses frequently require hospitalization for workup and initiation of treatment. Treatment involves prompt diagnosis, long-term antimicrobial therapy, and sometimes surgical debridement. Though there is a lack of universally-accepted diagnostic criteria for these infections, a combination of pathogen detection, imaging consistent with osteomyelitis and/or septic arthritis, and clinical data such as inability to bear weight, decreased range of motion, presence of fever, and elevated inflammatory markers or white blood cell (WBC) count is often used to confirm the diagnosis.

Pathogen detection is important to direct and narrow antimicrobial therapy, but patients may be treated for these infections even with negative cultures to avoid complications related to untreated infection. Cultures may not yield the causative organism, or the pathogen may only be isolated from the affected bone or joint [1, 5–7]. Rates of culture-negative pediatric musculoskeletal infection range from 16 to 42% [2, 8]. Initial blood cultures have been reported to be positive in 30–50% of children with acute hematogenous osteomyelitis [3, 8, 9], whereas bone biopsy cultures are reported to be positive in 50–70% [10, 11].. A systematic review and several other small studies of pediatric patients with acute osteomyelitis and/or septic arthritis found that pathogens were more likely to be isolated from culture samples at the source of infection (joint aspirate, bone biopsy, pus) than from blood culture [1, 9, 12–14].

Though empiric antibiotics are the mainstay of treatment, the ideal time to initiate antibiotics is debated. Empiric antibiotics may decrease pathogen isolation thereby preventing targeted antimicrobial treatment, whereas delay in treatment may lead to increased complications. The optimal time to initiate antibiotics to prevent treatment delays while maximizing culture yield is not well studied in the pediatric population, and existing studies tend to be small. Several pediatric studies have found no difference in operative culture results between patients whose cultures were pretreated with antibiotics and those who did not receive antibiotics, though there was an association between longer duration of antibiotics and likelihood of having a negative culture [8, 15–17]. Identifying the effect of antibiotics on culture yield may help clinicians determine when to initiate antimicrobial therapy.

In this study, we sought to examine associations between laboratory values, duration of antibiotics prior to culture, and culture type on pathogen identification of blood, bone, joint, and “other” (wound, abscess, or fluid other than joint fluid) bacterial cultures. To our knowledge, this is the largest study performed to evaluate culture type and duration of antibiotic pretreatment on culture results in pediatric osteomyelitis and/or septic arthritis.

## Methods

### Study design

This was a retrospective cohort study of children between 30 days and 18 years of age admitted to two freestanding tertiary children’s hospitals within the same institution in the Southeastern United States with acute hematogenous osteomyelitis and/or septic arthritis over an 8-year period between January 2009 and December 2016. The study was approved by our hospital’s Institutional Review Board.

### Selection of participants

Patients were included if they were treated for osteomyelitis and/or septic arthritis based on clinical documentation. Patients were excluded if ultimate management and treatment were not consistent with osteomyelitis or septic arthritis based off imaging findings, cultures, and treatment course (e.g. pyomyositis alone). Patients were also excluded if they had recent trauma such as fracture or foreign body (minor trauma such as bumps or bruises noted in history were still included), had post-operative infection or hardware at the site of the infection, had chronic symptoms (> 6 weeks in duration), had chronic recurrent multifocal osteomyelitis (CRMO), were immunocompromised (e.g. malignancy, sickle cell, chronic immunosuppressive or immunomodulator therapy, transplant recipient, or primary immune disorder), if they were a neonate who had never left the hospital, or if they had the majority of their workup at an outside hospital.

### Data collection

Subjects were identified via International Classification of Diseases, Ninth Revision and Tenth Revision (ICD-9 and ICD-10) queries of our electronic medical record (EMR), Epic. We included the ICD-9 codes 730, 730.2, 730.3, and 730.9 for osteomyelitis and 711.0 and 711.9 for pyogenic arthritis, as well as the ICD-10 codes M86.9 for osteomyelitis and M00.9 for pyogenic arthritis. Charts of patients identified via these queries were reviewed for admission history and physical, progress notes, subspecialty consult notes, and discharge summaries to determine if the patient was treated for osteomyelitis and/or septic arthritis. Patients were included if they were treated for one of these diagnoses and excluded if these diagnoses were clinically ruled out. Data collected on these patients included socio-demographics, presenting symptoms, timing of antibiotics, laboratory results, and results of microbiologic and radiographic studies. Data were entered into Research Electronic Data Capture (REDCap, Vanderbilt University, Nashville TN), a secure web application for managing databases, and were de-identified. The type of culture (blood, joint aspirate, bone, “other”) was based off the specimen type recorded in the EMR. “Other” cultures were cultures from wound, abscess, or fluid other than joint fluid collected from the suspected site of infection (eg. subperiosteal abscess) in patients with other clinical, laboratory, or radiographic evidence of osteomyelitis and/or septic arthritis. Time to initiation of antibiotics was defined as the time of admission in our EMR to the time of the first documented administration of an antibiotic. Time to culture was defined as the time of admission in our EMR to the time the culture was obtained. Culture positivity was defined as having a culture which grew a pathogen and pretreated cultures were defined as having been exposed to antibiotics as an outpatient, in an outside emergency room, or at our institution, within 1 week of admission, prior to cultures being obtained. Duration of antibiotic exposure was defined as time of the first dose of antibiotics to the time the culture was obtained. If documentation stated a definitive number of days the patient had received antibiotics instead of exact timing, we presumed those were full days of treatment.

### Statistical analysis

All statistical analyses were performed using SAS 9.4 (Carey, North Carolina). Descriptive statistics were calculated for variables of interest, including medians and interquartile ranges (25th and 75th percentiles) for continuous variables, and counts and percentages for categorical variables. Logistic regression was used to assess the effect of antibiotic pretreatment and duration on culture positivity. All models were adjusted for age, gender, race, days of illness, and C-reactive protein. Odds ratios with 95% confidence intervals were presented. A *p*-value of < 0.05 assumed statistical significance.

### Outcome measures

Primary outcome was culture positivity. Symptoms and laboratory values at presentation are described and were used to attempt to control for illness severity. The effect of antibiotic duration on culture positivity was also analyzed.

## Results

### Characteristics of the study sample

**Fig. 1:**
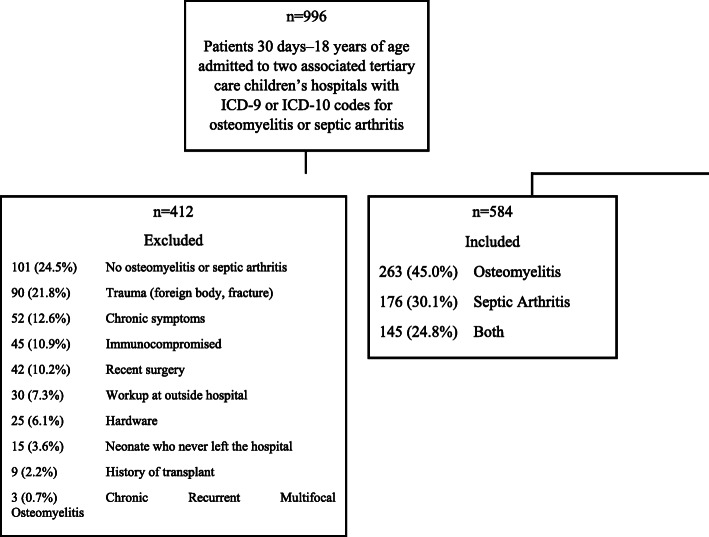
Study Population

Out of 996 unique charts which had an ICD-9 or ICD-10 code for osteomyelitis or septic arthritis, 584 patients (58.6%) met inclusion criteria (Fig. 1). Of these patients, 263 (45.0%) had only osteomyelitis, 176 (30.1%) had only septic arthritis, and 145 (24.8%) had both osteomyelitis and septic arthritis. A total of 521 of the 584 (90%) included patients had an MRI that was used to help confirm the diagnosis, including 252 of 263 (96%) patients with osteomyelitis, 131 of 176 patients (74%) with septic arthritis, and 138 of 145 (95%) patients with both osteomyelitis and septic arthritis. Of the 219 patients who had negative cultures, 194 (89%) received an MRI which was used to help confirm the diagnosis which was similar to patients who had at least one positive culture (327/365, 90%). Demographics and clinical data of the included patients are displayed in Table 1.

**Table 1:** Characteristics of the Study Sample

Characteristics^**a**^	All Subjects (***n*** = 584)
Age, years	6.1 (1.9–11.0)
Male	60.6%
Race/Ethnicity	
Caucasian	53.8%
African-American	32.7%
Asian	4.3%
Hispanic	1.5%
Other/Unknown	7.7%
Race, collapsed	
White vs non-white	53.8%
Duration of symptoms at presentation, days	4.0 (3.0–7.0)
Fever ≥38 °C at presentation	79.0%
Initial WBC^b^ count (×10^9^/L)	11.6 (8.8–15.4)
Initial ESR^c^ (mm/h)	45.0 (28.0–66.0)
Initial CRP^d^ (mg/dL)	6.7 (3.0–16.0)

^a^Results are presented as median [interquartile range (IQR)] or %

^b^WBC = white blood cell

^c^ESR = erythrocyte sedimentation rate

^d^CRP = C-reactive protein

*Missing Values: WBC (n = 3, 0.5% of total data); ESR (n = 71, 12.2%); CRP (n = 7, 1.2%)*

Overall, median age was 6.1 years, 60.6% were male, and 53.8% were Caucasian. Children who had pretreated bone cultures were more often Caucasian (62.7% vs 46.2%, *p* = 0.04). Reported symptoms showed a median of 4 days of symptoms at the time of presentation and 79.0% had a history of fever ≥38 °C. Children with pretreated blood or bone cultures were more likely to have fever at presentation than those whose blood or bone cultures were not pretreated (89.6% vs 77.8%, *p* = 0.003 for blood cultures; 86.3% vs 69.2%, *p* = 0.05 for bone cultures). Initial labs showed a median WBC count of 11.6 × 10^9^/L, median erythrocyte sedimentation rate (ESR) of 45.0 mm/h, and median C-reactive protein (CRP) of 6.7 mg/dL. For all culture types, patients whose cultures were pretreated had a higher initial CRP (12.4 vs 5.9, *p* < 0.001 in blood; 12.8 vs 4.2, *p* = 0.002 in bone; 12.6 vs 5.2, p < 0.001 in joint; 11.6 vs 5.9, *p* = 0.008 in “other”). Subgroup analysis data is not shown (see Additional File 1: Subgroup Analysis of Table 1).

### Impact of antibiotics on culture yield

Blood cultures were obtained in 525 patients (89.9%), bone cultures in 90 patients (15.4%), joint cultures in 265 patients (45.4%), and “other” cultures in 197 patients (33.7%). Patients were treated with antibiotics prior to culture in 125 of 525 (23.8%) blood cultures, 51 of 90 (56.7%) bone cultures, 92 of 265 (34.7%) joint cultures, and 140 of 197 (71%) “other” cultures. There was no significant difference in culture positivity between cultures that were pretreated with antibiotics and those that were not pretreated (Table 2). Post-hoc power analysis showed we could detect effect size differences by antibiotic pretreatment of 0.29, 0.60, 0.36, and 0.44 in blood, bone, joint, and “other” culture positivity, respectively. There are moderate differences for bone and “other” cultures (observed effect sizes are shown in Table 2).

**Table 2:** Percent of Positive Result, Overall and by Pretreatment Status

Culture Type	N	Rate of Culture Positivity				
		Overall (%)	Pretreated (%)	Not Pretreated (%)	P-value	Effect Size
Blood Culture	525	221 (42%)	55 (44%)	166 (42%)	0.62	0.04
Bone Culture	90	63 (70%)	38 (75%)	25 (64%)	0.29	0.25
Joint Culture	265	103 (39%)	36 (39%)	67 (39%)	0.95	0.01
Other Culture	197	138 (70%)	103 (74%)	35 (61%)	0.09	0.26

P-value: chi-squared test of independence between pretreated and not pretreated groups

Effect size: standardized mean difference between pretreated vs. not pretreated groups

To determine if antibiotic pretreatment or duration was associated with a change in culture positivity, logistic regression models were used to adjust for age, gender, and race. In an attempt to control for illness severity, these models also adjusted for days of illness and CRP (Table 3). Patients without all variables were excluded from logistic regression analysis (*n* = 7, 1.2% of the total dataset). Blood, bone, and joint cultures that were pretreated with antibiotics had lower odds of a positive culture compared to cultures that were not pretreated, but results were not statistically significant (Table 3). Using categorized hours of antibiotic duration as a predictor, there was no significant association between patients receiving antibiotics for 0 to < 4 h, 4 to < 24 h, or ≥ 24 h for any of the pretreated cultures and the odds of a positive culture result (Table 3).

**Table 3:** Logistic Regression Models, Association of Antibiotic Pretreatment and Duration on Culture Positivity

Predictor	Culture	Comparison (reference = no pretreatment)	Odds Ratio for Positive Culture	
**Pretreatment (vs. none) with antibiotics**			**OR**^**a**^**(95% CI**^**b**^**)**	**P-value**
*N* = 525	Blood	–	0.90 (0.56–1.44)	0.66
*N* = 90	Bone	–	0.77 (0.25–2.34)	0.64
*N* = 262	Joint	–	0.71 (0.39–1.28)	0.26
*N* = 196	Other	–	1.18 (0.58–2.41)	0.65
**Hours of antibiotics (for pretreated cultures)**	**Culture**	**Comparison** **(reference = 0- < 4 h)**	**OR (95% CI)**	**P-value**
*N* = 113	Blood	4 to < 24 h	1.24 (0.44–3.52)	0.90
		> 24 h	1.34 (0.35–5.22)	0.78
*N* = 50	Bone	4 to < 24 h	1.11 (0.06–19.93)	0.90
		> 24 h	1.57 (0.08–31.4)	0.68
*N* = 91	Joint	4 to < 24 h	1.36 (0.36–5.06)	0.93
		> 24 h	1.67 (0.35–8.01)	0.58
*N* = 139	Other	4 to < 24 h	0.77 (0.24–2.46)	0.28
		> 24 h	1.60 (0.44–5.77)	0.25

Note: All logistic regression models adjusted for age, gender, race, duration of symptoms at presentation, and C-reactive protein

^a^OR: Odds Ratio

^b^CI: Confidence Interval

## Discussion

Rates of culture positivity in this study were in keeping with prior studies [6, 8, 12, 14, 15]. Antimicrobial data are described separately [18]. There was no association between culture positivity and pretreatment with antibiotics in any of our culture samples. The moderate effect sizes seen for bone and “other” cultures suggests that these results could be potentially found as statistically significant with a reasonably larger sample size. Furthermore, the duration of antibiotics prior to culture did not affect culture yield for any culture type, even after controlling for factors such as age, gender, race, day of illness, and CRP. This finding is in contrast to a prior study which showed that among patients who were given antibiotics prior to culture, those with positive cultures had a shorter median duration of pretreatment than those with negative cultures, though they did not reach statistical significance [14]. We hypothesized that patients who appeared more ill at presentation would be more likely to receive antibiotics prior to culture, which is corroborated by the patients whose cultures were pretreated having a higher initial CRP than those whose cultures were not pretreated, though we did attempt to control for illness severity by adjusting for CRP in the logistic regression models. Since there is no gold standard for the diagnosis of osteomyelitis and/or septic arthritis without positive cultures, our study included patients who were treated for these diagnoses based on clinical evaluation and judgement even when cultures were negative. Therefore, patients who did not have osteomyelitis and/or septic arthritis may have been included in our analysis which would have had a dilutional effect on the culture-negative group, biasing our results toward the null.

Acute osteomyelitis and septic arthritis are two pediatric diagnoses which frequently require hospitalization for initial management. Timely diagnosis and appropriate treatment may minimize complications and optimize outcomes. Prompt diagnosis and treatment may help prevent systemic disease or lifelong musculoskeletal deformities such as disruption of longitudinal bone growth, joint destruction, growth failure, sepsis, or death. One systematic review found that delay in treatment was a risk factor for poor prognosis, citing several studies with a decrease in cure rate when treatment was delayed [1].

There are also concerns that initiating antibiotic treatment prior to obtaining cultures decreases culture yield. Studies examining the impact of antibiotic treatment on culture results in pediatric musculoskeletal information have mixed results [8, 15–17]. The optimal time to initiate antibiotics to prevent treatment delays and complications while maximizing culture yield is not well studied. To our knowledge, this is the largest study to date examining the effect and timing of antibiotics on culture results in this population.

### Limitations

There were several limitations to the study. This study was retrospective, so we may not have a comprehensive database of all cultures obtained in musculoskeletal infections. Approximately 10% of the initial charts were excluded due to not having septic arthritis or osteomyelitis which underscores that using ICD-9 and ICD-10 has a risk of diagnosis misclassification. Patients who are initially treated for presumed osteomyelitis and/or septic arthritis may ultimately have these diagnoses ruled out clinically. Using these codes also has a risk of missing patients who truly presented with osteomyelitis and/or septic arthritis. Our definition of pretreatment relied on the provided documentation which was sometimes not specific. Reported histories also introduces the potential for recall bias. The data available in our retrospective review did not allow us to account for other factors which may have contributed to culture positivity (eg. swab vs. syringe, culture bottle vs. plates, etc). While our study had a relatively large sample size, some of the subgroups were small, but the post-hoc power analysis did show moderate effect sizes for bone and “other” cultures. The retrospective nature of our study design does not allow for definitive associations between antibiotic pretreatment and culture results. Lastly, we had no method to account for the degree of illness, though we attempted to mitigate this confounder by controlling for days of illness and CRP.

## Conclusions

Pretreatment of patients with antibiotics prior to culture was not associated with a significant change in culture yield, even as the duration of antibiotics increased. Routinely initiating antibiotics early in the illness course may enable reduction of variability and improve coordination of care as these infections often require a multidisciplinary approach. Prospective studies or trials are needed to determine if early initiation of antibiotics affects culture positivity or improves clinical outcomes.

## Supplementary Information

**Additional file 1.** Subgroup Analysis of Table 1: Characteristics of the Study Sample by Pre-treatment with Antibiotics before Culture.

## Data Availability

The datasets generated and/or analyzed during the current study are not publicly available. These data are the property of the Children’s Healthcare of Atlanta but are available from the corresponding author upon reasonable request.

## Abbreviations

CRP: C-reactive protein
CRMO: chronic recurrent multifocal osteomyelitis
EMR: Electronic medical record
ESR: Erythrocyte sedimentation rate
ICD-9: International Classification of Diseases, 9th Revision
ICD-10: International Classification of Diseases, 10th Revision
REDCap: Research Electronic Data Capture
WBC: White blood cell
