# Supplementary material for: Impact of antibiotic pretreatment on cultures in children with osteomyelitis and septic arthritis: a retrospective review
Source: BMC Pediatr. 2021 Aug 13;21:342. doi: 10.1186/s12887-021-02806-w (PMC8361620; doi:10.1186/s12887-021-02806-w)
Supplement: Supplementary file 1 — Additional file 1. Subgroup Analysis of Table 1: Characteristics of the Study Sample by Pre-treatment with Antibiotics before Culture. [file 12887_2021_2806_MOESM1_ESM.docx]

**Impact of Antibiotic Pretreatment on Cultures in Children with Osteomyelitis and Septic Arthritis: A Retrospective Review**

Amanda Lansell,^1,2,3^ MD, Yasasvi Vasili,^2^ MD, Parminder S. Suchdev,^1,2^ MD, MPH, Janet Figueroa,^2^ MPH, Anjali Kirpalani,^1,2^ MD

**Affiliations:** ^1^Children’s Healthcare of Atlanta, Atlanta, Georgia; ^2^Emory University Department of Pediatrics, Atlanta, Georgia; ^3^Rainbow Babies and Children’s Hospital, Cleveland, Ohio

**Address correspondence to:** Amanda Lansell, Department of Pediatrics, Rainbow Babies and Children’s Hospital, 11100 Euclid Avenue, Cleveland OH, 44106 (current affiliation), [alansell22@gmail.com], 330-428-2321

*Affiliation where research took place*: Children’s Healthcare of Atlanta, 1405 Clifton Rd, Atlanta GA, 30329

**Subgroup Analysis of Table 1: Characteristics of the Study Sample by Pre-treatment with Antibiotics before Culture**

| **Characteristics^1^** | **All Subjects in Study**  **(*n* = 584)** | **Not Pretreated with Antibiotics**  **N=400** | **Pretreated with Antibiotics**  **N=125** | **P-value^2^** | **SD^3^** |
| --- | --- | --- | --- | --- | --- |
| **Blood Culture** |  |  |  |  |  |
| Age, years | 6.1 (1.9-11.0) | 5.9 (1.6-10.9) | 6.2 (2.8-11.3) | 0.22 | 0.13 |
| Male | 60.6% | 60.0% | 64.8% | 0.34 | 0.10 |
| Race |  |  |  | 0.31 | 0.23 |
| Caucasian | 53.8% | 51.8% | 60.8% |  |  |
| African-American | 32.7% | 35.5% | 27.2% |  |  |
| Asian | 4.3% | 4.5% | 2.4% |  |  |
| Hispanic | 1.5% | 1.0% | 1.6% |  |  |
| Other | 7.7% | 7.3% | 8.0% |  |  |
| Race, collapsed |  |  |  | 0.08 | 0.18 |
| White vs non-white | 53.8% | 51.8% | 60.8% |  |  |
| Type |  |  |  | 0.20 | 0.18 |
| Osteomyelitis Only | 45.0% | 47.5% | 46.4% |  |  |
| Septic Arthritis Only | 30.1% | 29.3% | 23.2% |  |  |
| Both | 24.8% | 23.3% | 30.4% |  |  |
| Duration of symptoms at  presentation, days | 4.0 (3.0-7.0) | 4.0 (2.0-7.0) | 4.0 (3.0-7.0) | 0.14 | 0.15 |
| Fever at presentation | 79.0% | 77.8% | 89.6% | 0.003 | 0.29 |
| Initial WBC^4^ count (x10^9^/L) | 11.6 (8.8-15.4) | 11.4 (8.7-14.7) | 12.2 (8.3-16.8) | 0.22 | 0.12 |
| Initial ESR^5^ (mm/h) | 45.0 (28.0-66.0) | 44 (28-65.5) | 50.0 (32.0-74.0) | 0.06 | 0.21 |
| Initial CRP^6^ (mg/dL) | 6.7 (3.0-16.0) | 5.9 (2.9-14.6) | 12.4 (4.6-20.0) | <0.001 | 0.40 |
| Length of Stay (days) | 5.8 (4.6-8.1) | 5.7 (4.5-8.1) | 6.7 (5.2-9.7) | 0.005 | 0.29 |
| ^1^Results are presented as median [interquartile range (IQR)] or %  ^2^Wilcoxon Rank-sum tests or Fisher’s/Chi-squared tests comparing Pre-Treated and Not Pre-Treated groups  ^3^SD = standardized (rank-based) difference (effect size)  ^4^WBC = white blood cell  ^5^ESR = erythrocyte sedimentation rate  ^6^CRP = C-reactive protein | | | | | |

| **Characteristics^1^** | **Not Pretreated with Antibiotics**  **N=39** | **Pretreated with Antibiotics**  **N=51** | **P-value^2^** | **SD^3^** |
| --- | --- | --- | --- | --- |
| **Bone Culture** |  |  |  |  |
| Age, years | 5.9 (1.5-11.3) | 7 (3.9-11.6) | 0.22 | 0.26 |
| Male | 71.8% | 58.8% | 0.20 | 0.28 |
| Race |  |  | 0.04 | 0.63 |
| Caucasian | 46.2% | 62.7% |  |  |
| African-American | 43.6% | 33.3% |  |  |
| Asian | 10.3% | 0 |  |  |
| Hispanic | 0 | 0 |  |  |
| Other | 0 | 3.9% |  |  |
| Race, collapsed |  |  | 0.12 | 0.34 |
| White vs non-white | 46.2% | 62.7% |  |  |
| Type |  |  | 0.98 | 0.04 |
| Osteomyelitis Only | 59% | 60.8% |  |  |
| Septic Arthritis Only | 7.7% | 7.8% |  |  |
| Both | 33.3% | 31.4% |  |  |
| Duration of symptoms at  presentation, days | 6.0 (3.0-8.0) | 5.0 (3.0-8.0) | 0.65 | 0.10 |
| Fever at presentation | 69.2% | 86.3% | 0.05 | 0.34 |
| Initial WBC^4^ count (x10^9^/L) | 9.5 (7.9-11.7) | 11.6 (9.2-15.7) | 0.01 | 0.56 |
| Initial ESR^5^ (mm/h) | 42.0 (20.0-66.0) | 55.0 (32.0-79.0) | 0.10 | 0.37 |
| Initial CRP^6^ (mg/dL) | 4.2 (1.5-10.4) | 12.8 (4.3-19.3) | 0.002 | 0.68 |
| Length of Stay (days) | 5.6 (4.6-9) | 7.1 (5-11.1) | 0.09 | 0.37 |
| ^1^Results are presented as median [interquartile range (IQR)] or %  ^2^Wilcoxon Rank-sum tests or Fisher’s/Chi-squared tests comparing Pre-Treated and Not Pre-Treated groups  ^3^SD = standardized (rank-based) difference (effect size)  ^4^WBC = white blood cell  ^5^ESR = erythrocyte sedimentation rate  ^6^CRP = C-reactive protein | | | | |

| **Characteristics^1^** | **Not Pretreated with Antibiotics**  **N=173** | **Pretreated with Antibiotics**  **N=92** | **P-value^2^** | **SD^3^** |
| --- | --- | --- | --- | --- |
| **Joint Culture** |  |  |  |  |
| Age, years | 4.3 (1.5-9.7) | 5.8 (2-9.3) | 0.39 | 0.11 |
| Male | 60.7% | 63.0% | 0.71 | 0.05 |
| Race |  |  | 0.39 | 0.29 |
| Caucasian | 56.6% | 56.5% |  |  |
| African-American | 26.6% | 30.4% |  |  |
| Asian | 6.4% | 1.1% |  |  |
| Hispanic | 1.7% | 2.2% |  |  |
| Other | 8.7% | 9.8% |  |  |
| Race, collapsed |  |  | 0.98 | <0.01 |
| White vs non-white | 56.6% | 56.5% |  |  |
| Type |  |  | 0.19 | 0.24 |
| Osteomyelitis Only | 11.6% | 12% |  |  |
| Septic Arthritis Only | 57.8% | 46.7% |  |  |
| Both | 30.6% | 41.3% |  |  |
| Duration of symptoms at  presentation, days | 3.0 (2.0-6.0) | 4.0 (2.0-6.0) | 0.74 | 0.04 |
| Fever at presentation | 75.7% | 84.8% | 0.09 | <0.01 |
| Initial WBC^4^ count (x10^9^/L) | 11.7 (9.4-14.9) | 12.5 (9.1-16.8) | 0.42 | 0.10 |
| Initial ESR^5^ (mm/h) | 44.0 (28.0-66.0) | 52.0 (35.0-76.0) | 0.04 | 0.28 |
| Initial CRP^6^ (mg/dL) | 5.2 (2.5-13.7) | 12.6 (4.7-20.9) | <0.001 | 0.49 |
| Length of Stay (days) | 5.1 (4-7.6) | 6.7 (4.9-10.3) | <0.001 | 0.47 |
| ^1^Results are presented as median [interquartile range (IQR)] or %  ^2^Wilcoxon Rank-sum tests or Fisher’s/Chi-squared tests comparing Pre-Treated and Not Pre-Treated groups  ^3^SD = standardized (rank-based) difference (effect size)  ^4^WBC = white blood cell  ^5^ESR = erythrocyte sedimentation rate  ^6^CRP = C-reactive protein | | | | |

| **Characteristics^1^** | **Not Pretreated with Antibiotics**  **N=57** | **Pretreated with antibiotics**  **N=140** | **P-value^2^** | **SD^3^** |
| --- | --- | --- | --- | --- |
| **Other Culture** |  |  |  |  |
| Age, years | 5.4 (1.6-10.5) | 6 (1.8-10.9) | 0.50 | 0.11 |
| Male | 56.1% | 67.1% | 0.15 | 0.23 |
| Race |  |  | 0.65 | 0.29 |
| Caucasian | 52.6% | 50% |  |  |
| African-American | 36.8% | 38.6% |  |  |
| Asian | 3.5% | 2.9% |  |  |
| Hispanic | 0 | 3.6% |  |  |
| Other | 7% | 5% |  |  |
| Race, collapsed |  |  | 0.74 | 0.05 |
| White vs non-white | 52.6% | 50% |  |  |
| Type |  |  | 0.26 | 0.25 |
| Osteomyelitis Only | 36.8% | 44.3% |  |  |
| Septic Arthritis Only | 31.6% | 20.7% |  |  |
| Both | 31.6% | 35.0% |  |  |
| Duration of symptoms at  presentation, days | 4.0 (3.0-7.0) | 4.0 (3.0-7.0) | 0.77 | 0.05 |
| Fever at presentation | 77.2% | 82.1% | 0.43 | 0.05 |
| Initial WBC^4^ count (x10^9^/L) | 11.2 (8.3-14.2) | 12.5 (9.2-17.2) | 0.07 | 0.31 |
| Initial ESR^5^ (mm/h) | 45.0 (31.0-70.0) | 50.0 (30.0-68.0) | 0.94 | 0.01 |
| Initial CRP^6^ (mg/dL) | 5.9 (3.1-10.4) | 11.6 (3.7-21.7) | 0.008 | 0.44 |
| Length of Stay (days) | 5.0 (4.2-7.8) | 6.4 (5.0-9.9) | 0.02 | 0.38 |
| ^1^Results are presented as median [interquartile range (IQR)] or %  ^2^Wilcoxon Rank-sum tests or Fisher’s/Chi-squared tests comparing Pre-Treated and Not Pre-Treated groups  ^3^SD = standardized (rank-based) difference (effect size)  ^4^WBC = white blood cell  ^5^ESR = erythrocyte sedimentation rate  ^6^CRP = C-reactive protein | | | | |
